# Next-generation PET/CT imaging in meningioma—first clinical experiences using the novel SSTR-targeting peptide [^18^F]SiTATE

**DOI:** 10.1007/s00259-023-06315-z

**Published:** 2023-06-26

**Authors:** Marcus Unterrainer, Sophie C. Kunte, Lena M. Unterrainer, Adrien Holzgreve, Astrid Delker, Simon Lindner, Leonie Beyer, Matthias Brendel, Wolfgang G. Kunz, Michael Winkelmann, Clemens C. Cyran, Jens Ricke, Klaus Jurkschat, Carmen Wängler, Björn Wängler, Ralf Schirrmacher, Claus Belka, Maximilian Niyazi, Joerg-Christian Tonn, Peter Bartenstein, Nathalie L. Albert

**Affiliations:** 1 Department of Nuclear Medicine, University Hospital, LMU Munich, Munich, Germany; 2 Department of Radiology, University Hospital, LMU Munich, Marchioninistr. 15, 81377 Munich, Germany; 3 German Center for Neurodegenerative Diseases (DZNE), Munich, Germany; 4 Munich Cluster for Systems Neurology (SyNergy), Munich, Germany; 5 Fakultät für Chemie und Chemische Biologie, Technische Universität Dortmund, Dortmund, Germany; 6 Biomedical Chemistry, Department of Clinical Radiology and Nuclear Medicine, Medical Faculty Mannheim of Heidelberg University, Mannheim, Germany; 7 Molecular Imaging and Radiochemistry, Department of Clinical Radiology and Nuclear Medicine, Medical Faculty Mannheim of Heidelberg University, Mannheim, Germany; 8 Department of Oncology, Division of Oncological Imaging, University of Alberta, Edmonton, AB Canada; 9 Department of Radiation Oncology, University Hospital, LMU Munich, Munich, Germany; 10 German Cancer Consortium (DKTK), Partner Site Munich, German Cancer Research Center (DKFZ), 69120 Heidelberg, Germany; 11 Bavarian Cancer Research Center (BZKF), Munich, Germany; 12 Department of Neurosurgery, University Hospital, LMU Munich, Munich, Germany

**Keywords:** Meningioma, Somatostatin receptor (SSTR), PET, SiTATE

## Abstract

**Background:**

Somatostatin-receptor (SSTR)-targeted PET/CT provides important clinical information in addition to standard imaging in meningioma patients. [^18^F]SiTATE is a novel, ^18^F-labeled SSTR-targeting peptide with superior imaging properties according to preliminary data. We provide the first [^18^F]SiTATE PET/CT data of a large cohort of meningioma patients.

**Methods:**

Patients with known or suspected meningioma undergoing [^18^F]SiTATE PET/CT were included. Uptake intensity (SUV) of meningiomas, non-meningioma lesions, and healthy organs were assessed using a 50% isocontour volume of interest (VOI) or a spherical VOI, respectively. Also, trans-osseous extension on PET/CT was assessed.

**Results:**

A total of 107 patients with 117 [^18^F]SiTATE PET/CT scans were included. Overall, 231 meningioma lesions and 61 non-meningioma lesions (e.g., post-therapeutic changes) were analyzed. Physiological uptake was lowest in healthy brain tissue, followed by bone marrow, parotid, and pituitary (SUV_mean_ 0.06 ± 0.04 vs. 1.4 ± 0.9 vs. 1.6 ± 1.0 vs. 9.8 ± 4.6; *p* < 0.001). Meningiomas showed significantly higher uptake than non-meningioma lesions (SUV_max_ 11.6 ± 10.6 vs. 4.0 ± 3.3, *p* < 0.001). Meningiomas showed significantly higher uptake than non-meningioma lesions (SUVmax 11.6±10.6 vs. 4.0±3.3, p<0.001). 93/231 (40.3%) meningiomas showed partial trans-osseous extension and 34/231 (14.7%) predominant intra-osseous extension. 59/231 (25.6%) meningioma lesions found on PET/CT had not been reported on previous standard imaging.

**Conclusion:**

This is the first PET/CT study using an ^18^F-labeled SSTR-ligand in meningioma patients: [^18^F]SiTATE provides extraordinary contrast in meningioma compared to healthy tissue and non-meningioma lesions, which leads to a high detection rate of so far unknown meningioma sites and osseous involvement. Having in mind the advantageous logistic features of ^18^F-labeled compared to ^68^Ga-labeled compounds (e.g., longer half-life and large-badge production), [^18^F]SiTATE has the potential to foster a widespread use of SSTR-targeted imaging in neuro-oncology.

## Introduction

Meningiomas represent the most common brain tumors [1, 2]. Standard clinical imaging consists of CT and MRI; however, there is an unmet clinical need for additional molecular imaging regarding clinical issues that cannot be answered by standard morphological imaging alone [3, 4], e.g., the evaluation of osseous involvement or the differentiation of post-therapeutic changes or scars from tumor remnants or recurrence. Therefore, somatostatin-receptor (SSTR)-targeted PET imaging has gained increasing clinical relevance in the diagnosis of meningioma patients in several clinical settings [1, 5, 6]. The standard ligands for SSTR-PET imaging are [^68^Ga]Ga-DOTATATE and [^68^Ga]Ga-DOTATOC, which are predominantly used for the imaging of neuroendocrine neoplasms [7]. These ligands are, however, labeled with ^68^Ga, which is accompanied by certain drawbacks such as dependency on costly ^68^Ge/^68^Ga-generators, small batch production of the ^68^Ga-radiopharmaceutical, a relatively short half-life, and, consequently, a smaller number of patient slots available. Furthermore, given its relatively high positron energy, ^68^Ga yields inferior image resolution compared to other isotopes used for PET imaging. [^18^F]SiTATE is a novel SSTR-targeting peptide that uses silicon fluoride acceptor (SiFA) radiochemistry based on a one-step ^19^F-^18^F isotopic exchange reaction [8] and has already been automated on a Scintomics GRP™ platform [9, 10]. As [^18^F]SiTATE has already shown very promising results in preliminary studies in patients with neuroendocrine tumors [11–13], we aimed at evaluating the imaging characteristics of this novel SSTR-targeting ligand in patients with meningioma.

## Materials and methods

### Patients

We included consecutive patients with known or suspected meningioma without any pretreatment, patients with suspected recurrence/tumor remnants after specific pretreatments, and patients with meningioma for radiotherapy planning. Patients were referred by their treating neurosurgeons or radiation oncologists. All patients gave written consent to undergo [^18^F]SiTATE PET/CT according to the regulations of the German Pharmaceuticals Act §13(2b). This study was performed in compliance with the principles of the Declaration of Helsinki and its subsequent amendments. The analysis of the data was approved by the institutional ethics board of LMU Munich (IRB 22-0353).

### PET/CT imaging

**Fig. 1:**
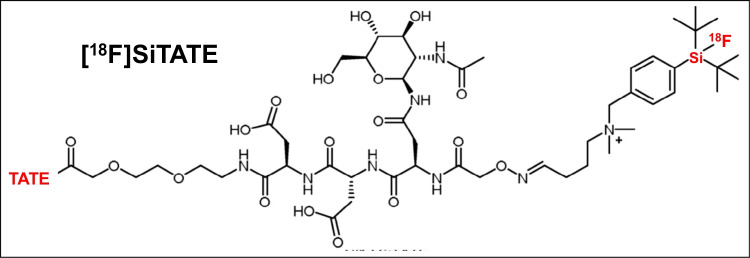
Structural formula [^18^F]SiTATE, see analogously [8]

SiTATE was obtained from ABX, Advanced Biomedical Compounds (Dresden, Germany). Radiosynthesis was performed at the Department of Nuclear Medicine, LMU Munich, Germany, as described previously [11, 14, 15]. All quality control measurements met the local product release criteria. After intravenous injection of [^18^F]SiTATE, PET scans were acquired at 90 min after injection for 15–20 min [11]. Additionally, the patients were premedicated with furosemide (20 mg/2 mL injection solution, ratiopharm GmbH, Ulm, Germany) for radiation protection, if no medical contraindication was given [16]. PET/CT included contrast-enhanced, diagnostic CT scans with 1.5 mL of iopromide (Ultravist-300, Bayer Healthcare, Leverkusen, Germany) per kilogram of body weight. No unusual symptoms or adverse effects after the tracer injection were noted. With CT scans serving for morphological correlation and attenuation correction, PET images were reconstructed with a transaxial 200 × 200 matrix using TrueX (including TOF, 2 iterations, and 21 subsets, and a 3D Gauss post-filter of 4-mm full width and a half maximum). All [^18^F]SiTATE PET/CT scans were acquired at the Department of Nuclear Medicine, LMU Munich, on a Siemens Biograph mCT flow or Siemens Biograph 64 (Siemens Healthineers, Erlangen, Germany) with a spatial resolution of 5.4 × 5.4 × 6.0 mm. The structural formula of [^18^F]SiTATE is presented in Fig. 1.

### Image analysis

Image analysis was performed using a dedicated software package (Hermes Hybrid Viewer, Affinity 1.1.4; Hermes Medical Solutions, Stockholm, Sweden). Background activity in healthy/unaffected organs (healthy brain, cervical bone marrow, parotid, and pituitary) was assessed using a 1 cm spherical volume of interest (VOI), where the mean/maximal standardized uptake value (SUV_mean_/_max_) was analyzed. Lesions, either suspicious for meningioma or non-meningioma, were analyzed using a 50% isocontour VOI approach or, in the case of very low uptake intensities, using a 2 cm spherical VOI, where SUV_max_ was noted, in order to exclude automatic inclusion of areas with physiological uptake. In the case of pituitary involvement, the respective VOI was refined manually. In a consensus read of three board-certified nuclear medicine physicians experienced with neuro-oncology imaging (MU, MB, and NLA), the extent of osseous involvement of meningiomas was rated as (I) no osseous extension, (II) partial trans-osseous extension, and (III) predominant intra-osseous extension.

In analogy to previous literature [17], we performed a standard clinical evaluation of meningioma PET/CT imaging. PET information was directly correlated to MRI and/or CT imaging. When obvious exceedance of dural and bone structures as defined by morphological imaging was present on PET, a partial trans-osseous involvement was noted. In the case of uptake on PET imaging restricted to the meningioma without any suspicion of extension to osseous structures, the scan was rated as extra-osseous meningioma. In the case of SSTR expression with overwhelmingly intra-osseous extension, the scan was rated as primarily intra-osseous meningioma. In the case of diverging estimations, a consensus of the 3 blinded readers was reached.

In every patient, all findings suggestive for meningioma sites on the PET/CT imaging were directly correlated with individual previous medical records and imaging reports to assess whether these lesions were already known or missed on previous standard morphological imaging. The PET/CT-based classification of meningioma or non-meningioma was performed using a consensus read including all PET/CT imaging findings, the medical record, consecutive or previous histological features, and follow-up imaging in the case where no histological samples were obtained in clinical routine.

### Statistical analysis

Data analysis was performed using Microsoft Excel (Excel 2019, Microsoft, Redmond, WA) and SPSS software (IBM SPSS Statistics 27, Armonk, NY). Descriptive statistics are displayed as median (range) or mean ± standard deviation (SD). Non-parametric Kruskal-Wallis test for unpaired and paired samples was used to determine the differences of continuous parameters. A two-tailed *p*-value  < 0.05 was considered statistically significant.

## Results

### Patient characteristics

A total of 107 patients with suspected meningioma or suspected meningioma recurrence were included (32 male patients (29.2%) and 75 female patients (70.8%)) with a mean age of 56.5 ± 16.7 years and underwent 117 PET/CT scans.

A histologically confirmed diagnosis was available in 70/107 patients (65.4%) in the previous disease course or after PET/CT imaging. Among these, 68/70 (97.1%) patients comprised histological verification of meningioma tissue (41/70 (58.5%) CNS WHO grade 1, 24/70 (34.3%) CNS WHO grade 2, and 3/70 (4.3%) CNS WHO grade 3). 2/70 (2.8%) patients comprised histologically confirmed non-meningioma lesions. The remaining patients showed characteristic imaging or clinical findings without further histological correlation.

In 40/117 (34.2%) cases, the patients did not undergo any tumor-specific therapy prior to the PET/CT scans or were under watch and wait strategy; in 74/117 (63.2%) cases, the patients had previous surgery; and in 20/117 cases (17.1%), patients underwent radiotherapy prior to PET/CT scanning. In only 3/117 (2.6%) scans, patients underwent systemic therapy, and in 2/117 (1.7%) cases, radioligand therapy was applied prior to PET/CT imaging. For further information, see Table 1.

**Table 1:** Patient characteristics

Parameters	
Patients (*n* = 107)	
Age	56.5 ± 16.7 years
Sex	
Male	32/107 (29.2%)
Female	75/107 (70.8%)
Histology available	70/107 (65.4%)
Non-meningioma	2/70 (2.8%)
CNS WHO grade 1 meningioma	41/70 (58.6%)
CNS WHO grade 2 meningioma	24/70 (34.3%)
CNS WHO grade 3 meningioma	3/70 (4.3%)
PET/CT scans (*n* = 117)	
Indications for PET/CT (multiple possible)	
Suspected meningioma/unclear lesion	27/117 (23.1%)
Suspected recurrence/follow-up	33/117 (28.2%)
Suspected remnant/exclusion remnant	21/117 (17.9%)
Radiotherapy planning	39/117 (33.3%)
Pretreatments prior to PET/CT (multiple possible)	
None/watch & wait	40/117 (34.2%)
Surgery	74/117 (63.2%)
Radiotherapy	20/117 (17.1%)
Targeted therapy	3/117 (2.6%)
Radioligand therapy	2/117 (1.7%)
Meningioma lesions (*n* = 231)	
No osseous involvement	104/231 (45.0%)
Partial trans-osseous extension	93/231 (40.3%)
Predominant intra-osseous extension	34/231 (14.7%)

### PET/CT imaging/indications

Overall, 117 PET/CT scans of 107 patients were included. A total of 156 ± 38MBq [^18^F]SiTATE was applied, no adverse events were noted, and all PET/CT scans comprised good image quality. Given the previously published radiation dose of approximately 0.015 mSv/MBq of [^18^F]SiTATE [11], the [^18^F]SiTATE PET scans included were accompanied by an approximate radiation exposure of 2.34 ± 0.57 mSv. Clinical indication for patients to undergo SSTR-targeted PET was suspected meningioma/suspicious lesion in 27/117 (23.1%) cases, suspected recurrence, or follow-up after specific therapies in 33/117 (28.2%) cases, suspected remnant/exclusion of remnants after surgery in 21/117 (17.9%) cases and radiotherapy planning in 39/117 cases (33.3%) (multiple indications possible, e.g., confirmation remnant and consecutive radiotherapy planning). 99/107 patients (92.6%) underwent only 1 PET/CT scan, 7/107 patients (6.5%) underwent 2 PET/CT scans, and 1/107 (0.9%) patients underwent 4 PET/CT scans.

### Uptake characteristics in healthy organs

In healthy organs, the lowest physiological uptake intensity was observed in healthy brain tissue/unaffected brain (SUV_mean_ 0.06 ± 0.04) followed by bone marrow (SUV_mean_ 1.42 ± 0.93) and uptake of the parotid gland (SUV_mean_ 1.64 ± 0.95); highest physiological uptake was noted in the pituitary gland (9.80 ± 4.58, *p* < 0.001) (see also Table 2).

**Table 2:** Uptake characteristics in healthy organs

Organ	Healthy brain	Bone marrow	Parotid gland	Pituitary gland	Level of significance
Uptake intensity SUV_mean_ [mean ± SD]	0.06 ± 0.04	1.42 ± 0.93	1.64 ± 0.95	9.80 ± 4.58	*p* < 0.001

### Uptake characteristics of meningioma and non-meningioma lesions

**Fig. 2:**
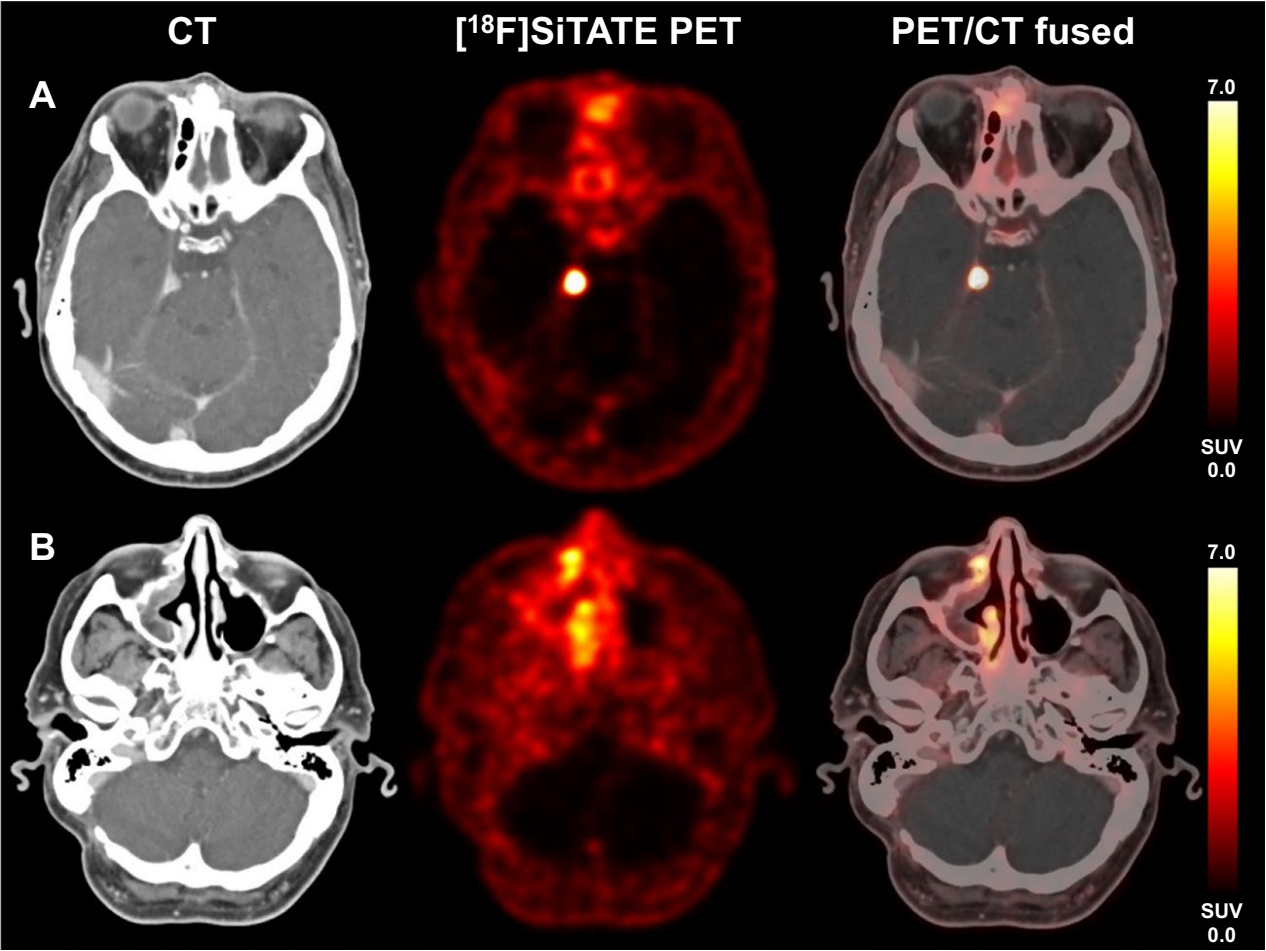
A 64-year-old with residual transitional meningioma, CNS WHO grade 1 at the right tentorium (**A**) with strong SSTR-expression (SUV_max_ 21.4), but also signs of chronic sinusitis at the right sinus maxillaris (**B**) with moderate SSTR-expression due to chronic inflammation (SUV_max_ 8.1)

Overall, 231 lesions were classified as meningioma sites, with a median number of 2 per patient (range, 0–12 lesions). These lesions comprised had a high mean uptake intensity (SUV_max_) of 12.6 ± 12.0. Moreover, 61 lesions/sites typical for non-meningioma findings/lesions were included. Non-meningioma sites were histologically confirmed in 2 patients; the remainder of the cases showed findings characteristic of reactive or inflammatory changes without any suspicion of meningioma involvement, e.g., post-operative changes at the surgery site and infectious sites such as acute sinusitis. Also, other SSTR-expressing lesions, e.g., corticotroph adenoma of the pituitary were included for comparison and classified as non-meningioma sites. These non-meningioma lesions/sites comprised a significantly lower mean SUV_max_ of 4.9 ± 4.1 (*p* < 0.001). Among these non-meningioma lesions, however, sites of high uptake intensity were also noted such as acoustic neuroma (SUV_max_ 8.6), sinusitis of the maxillary sinus (SUV_max_ 8.1), corticotroph adenoma (SUV_max_ 21.0), and degenerative changes of the facet joints (SUV_max_ 6.2). More information is displayed in Table 3 (see also Fig. 2).

**Table 3:** Uptake characteristics in meningioma and non-meningioma lesions

	Meningioma sites (*n* = 231)	Non-meningioma (*n* = 61)	Level of significance
Uptake intensity SUV_max_ [mean ± SD]	12.6 ± 12.0	4.9 ± 4.1	*p* < 0.001

### Pattern of osseous involvement and correlation with medical record

Among the 231 lesions classified as meningioma sites, 104/231 (45.0%) cases showed no osseous involvement. However, in 93/231 cases (40.3%), findings suggestive of trans-osseous extension on PET imaging were observed in a consensus read. Moreover, a predominant intra-osseous extension was noted in 34/231 cases (14.7%).

**Fig. 3:**
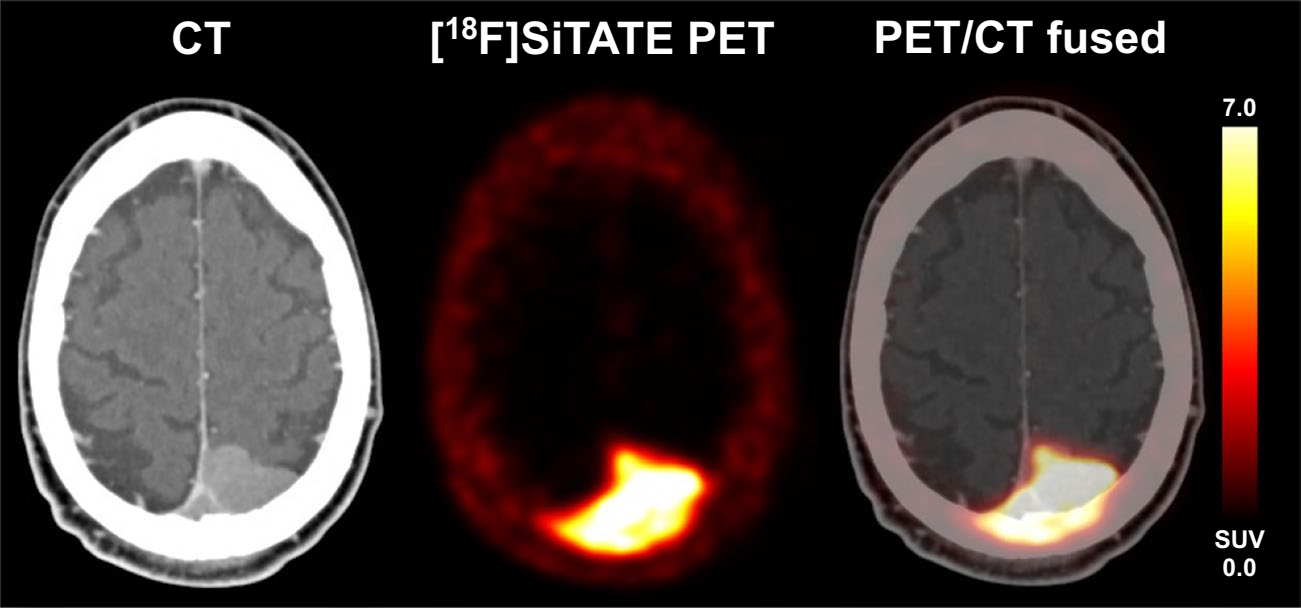
A case of a 51-year-old man with newly diagnosed occipital meningioma, which also showed infiltration of the super sagittal sinus with partial thrombosis and a trans-osseous extension within the occipital bone to the right side, which is easily detectable on [^18^F]SiTATE PET due to the strong SSTR expression (SUV_max_ 11.9). Resection revealed a CNS WHO grade 1 transitional meningioma

Each of the 231 lesions was directly compared to the documented medical record and imaging reports to assess whether each lesion was noted and reported in the previous medical history; here, 59/231 lesions were not reported on previous records and imaging reports, which accounts for 25.6% of all included meningioma sites (see also Fig. 3 for a patient example).

### Subgroup analysis—intra-individual comparison of [^18^F]SiTATE and [^68^Ga]Ga-DOTATOC

**Fig. 4:**
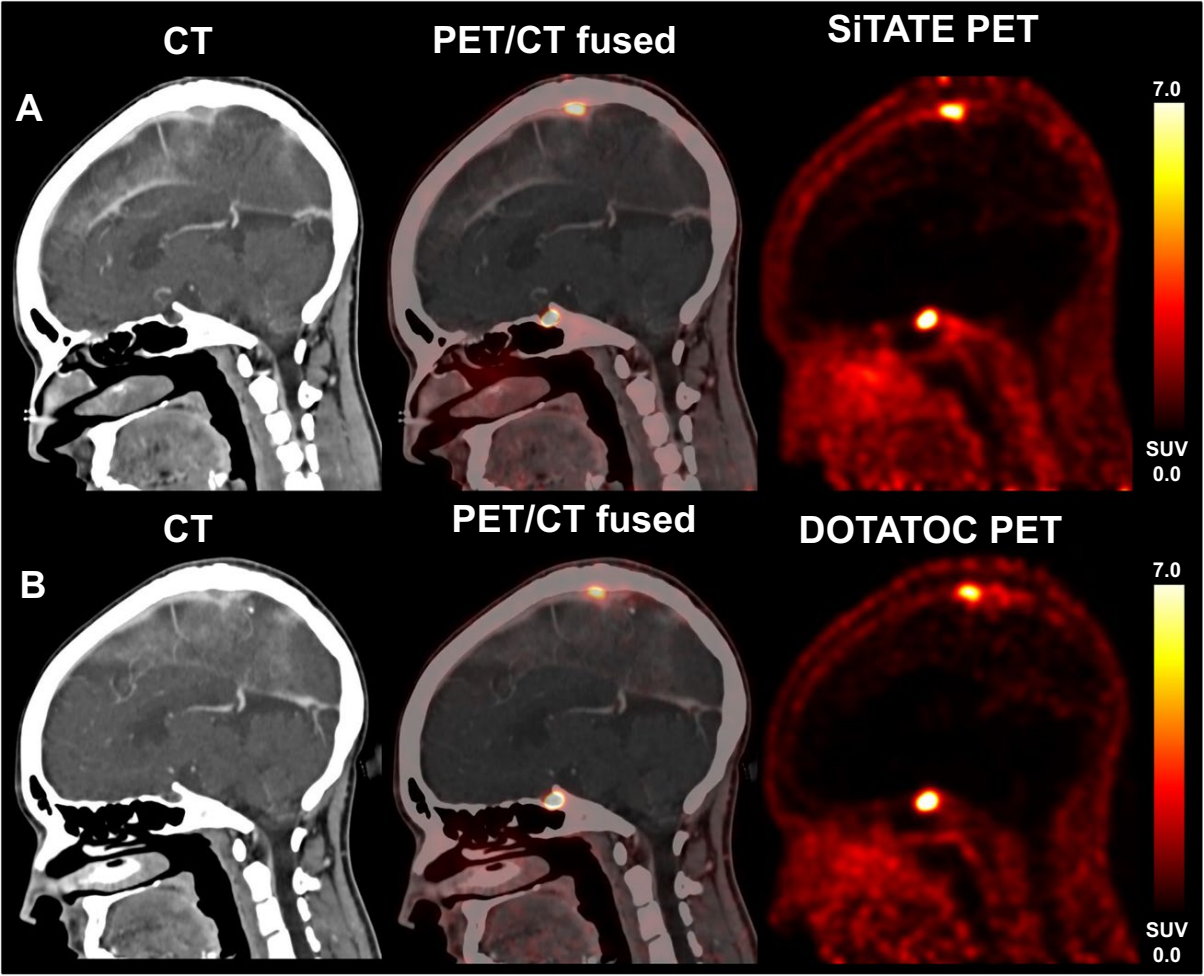
Follow-up imaging using [^18^F]SiTATE PET imaging: a patient with residual tumor remnant with extension to the superior sagittal sinus. The lesion at the superior sagittal sinus showed strong SSTR expression on [^18^F]SiTATE (**A**, SUV_max_ 8.6) without relevant changes in the course of 38 months without tumor-specific treatment. SSTR-expression on [^68^Ga]Ga-DOTATOC was comparable in the previous medical history (**B**, SUV_max_ 6.1)

In a subgroup of 7 patients with known meningioma without tumor-specific treatments, a previous [^68^Ga]Ga-DOTATOC PET/CT was available, where no relevant changes compared to the subsequent [^18^F]SiTATE PET/CT were noted after a median time of 24 months. Comparing the uptake intensity of healthy regions on [^18^F]SiTATE and [^68^Ga]Ga-DOTATOC PET, partially slightly higher SUV_mean_ values were noted using [^18^F]SiTATE compared to [^68^Ga]Ga-DOTATOC in the healthy brain (0.05 ± 0.02 vs. 0.03 ± 0.03, *p* = 0.034), the parotid (1.8 ± 0.5 vs. 1.8 ± 0.8, *p* = 0.310), the pituitary (10.7 ± 3.0 vs. 8.6 ± 3.4, *p* = 0.018), and the bone marrow (1.7 ± 0.6 vs. 0.8 ± 0.2, *p* = 0.091). In this subgroup, a mean activity of 204 ± 72 MBq was applied to [^68^Ga]Ga-DOTATOC PET imaging and a mean activity of 171 ± 53 MBq in the case of [^18^F]SiTATE PET. In the light of currently published radiation doses for these ligands (i.e., 0.015 mSv/MBq for [^18^F]SiTATE and 0.023 mSv/MBq for [^68^Ga]Ga-DOTATOC [11, 18]), this leads to a significantly lower radiation exposure when undergoing [^18^F]SiTATE PET imaging (2.6 ± 0.8 vs. 4.7 ± 1.7 mSv, *p* = 0.043) compared to [^68^Ga]Ga-DOTATOC (see Table 4). An exemplary case is presented in Fig. 4.

**Table 4:** Intra-individual comparison of uptake characteristics in patients with meningioma undergoing watch and wait without specific treatment (*n* = 7)

	Healthy brain [SUV_mean_]	Bone marrow [SUV_mean_]	Parotid gland [SUV_mean_]	Pituitary gland [SUV_mean_]	Meningiomas [SUV_max_]	Radiation dose [mSv]
[^18^F]SiTATE [mean ± SD]	0.05 ± 0.02	1.7 ± 0.6	1.8 ± 0.5	10.7 ± 3.0	6.8 ± 4.6	2.6 ± 0.8
[^68^Ga]Ga-DOTATOC [mean ± SD]	0.03 ± 0.03	0.8 ± 0.2	1.8 ± 0.8	8.6 ± 3.4	4.1 ± 2.0	4.7 ± 1.7
Level of significance	*p* = 0.034	*p* = 0.091	*p* = 0.310	*p* = 0.018	*p* = 0.116	*p* = 0.043

## Discussion

This is the first systematic analysis of the imaging characteristics of SSTR-directed PET/CT in patients with suspected or proven meningioma using [^18^F]SiTATE, a novel ^18^F-labeled SSTR-targeting ligand. In this exploratory analysis, we were able to investigate a large set of 107 patients with [^18^F]SiTATE PET, which is, moreover, one of the largest reported consecutive cohorts of meningioma patients undergoing PET imaging so far [19].

Patients were included in all clinical stages and for the current indications for SSTR-targeted PET imaging, e.g., radiotherapy planning or estimation of the osseous extent [1, 5]. In this regard, patients without or with pretreatments (radiotherapy, surgery, systemic therapies, or watch and wait strategy) were included, reflecting the whole range of clinical applications for SSTR-targeted PET imaging in meningioma. In a high percentage of patients, histological verification of meningioma tissue was available prior to or after the respective PET/CT scans, revealing the highest proportion of CNS WHO grade 1 meningiomas, followed by CNS WHO grade 2 and CNS WHO grade 3 meningiomas; however, the percentage of CNS WHO grade 2 meningiomas seems slightly overproportioned in the current cohort, most likely due to the higher clinical need of additional molecular imaging modalities, especially in challenging cases with unfavorable histological features [1, 20]. Given the clinical nature of (suspected) benign lesions or obviously post-therapeutic or reactive changes (e.g., hematoma or facet joint arthrosis), the number of non-meningioma lesions/sites with histological validation was low.

In line with previous reports in imaging with [^18^F]SiTATE in patients with neuroendocrine tumors [12, 13], no adverse events were noted after the application of [^18^F]SiTATE. In line with already established ^68^Ga-labeled ligands such as [^68^Ga]Ga-DOTATOC or [^68^Ga]Ga-DOTATATE, physiological uptake at unaffected areas was very low, with the lowest uptake in healthy brain tissue, most likely due to a missing blood-brain barrier permeability, followed by the parotid gland and bone marrow. As a result of the physiologically high SSTR expression of the pituitary [21], the significantly highest physiological uptake was noted in the pituitary.

Overall, tracer uptake of the 231 meningioma sites was high, with a mean SUV_max_ of 12.6 and uptake intensity quantifications up to an SUV_max_ of 90. In combination with the low tracer uptake in background tissues such as the healthy brain or the skull, these findings lead to a very high tumor-to-background contrast. For group comparison, we formed a control group of obvious or histologically verified non-meningioma lesions/sites such as post-operative changes after surgery, sinusitis, activated arthrosis, and hematoma, which do also comprise a certain degree of physiological or reactive SSTR expression, and, consecutively, uptake on SSTR-targeted PET imaging. When comparing these groups, a significantly higher uptake intensity in terms of SUV_max_ was noted in meningioma sites compared to non-meningioma sites, which underlines the capability of [^18^F]SiTATE for identifying meningioma sites due to the physiologically high SSTR expression of meningiomas and the relatively low uptake in reactive changes. However, there are also lesions at the meninges or the skull base, which comprise a physiologically high SSTR expression despite not being meningioma tissue, thus potentially mimicking meningioma on PET imaging. These cases have been extensively discussed using [^68^Ga]Ga-DOTATOC or [^68^Ga]Ga-DOTATATE, e.g., metastases of breast cancer, thyroid cancer, or nasopharyngeal cancer, but also granulomatous diseases such as neuro-sarcoidosis [22–26]. In the current set, some potential meningioma mimics were also noted, e.g., corticotroph adenoma of the pituitary, acoustic neuroma, or neuromuscular choristoma. Hence, knowledge of SSTR-expressing lesions beyond meningioma is essential to avoid over-calling of meningioma sites.

Osseous involvement has been acknowledged as an important finding with clinical implications, where SSTR-targeted PET is of help to assess the presence and extent of osseous involvement [5, 17, 27–29]. In the current consensus read, around 40% showed at least partial bone involvement, and around 15% of cases comprised a predominantly intra-osseous extension; these findings underline the ability of SSTR-targeted PET for evaluating bone invasion and the consecutive clinical implications for surgery or radiotherapy planning. Given the lower positron energy of ^18^F compared to ^68^Ga with a consecutively significantly higher spatial resolution of [^18^F]SiTATE compared to [^68^Ga]Ga-DOTATOC or [^68^Ga]Ga-DOTATATE, this physical property might also contribute to an improved and refined detection of bone involvement in meningioma patients. Of note, when comparing detected lesions suggestive of meningioma with the individual medical records and imaging reports, we found a surprisingly high proportion of lesions that had not been mentioned or noted within the previous medical history of around 25%. This finding must be interpreted in the context of the evaluated cohort of patients, who had been selected from the treating neurosurgeons or radiation oncologists to undergo PET imaging in addition to standard morphological imaging. Usually, meningioma patients are referred for SSTR-PET imaging when standard imaging cannot sufficiently answer the clinical issues, which is mostly the case for patients with meningioma with complex infiltration patterns, multilocular appearance, several pretreatments, and/or an unfavorable histological/molecular genetic profile. This might explain the high rate of small, undetected lesions given the skewed distribution towards more complex meningioma cases referred for PET imaging compared to “standard” benign meningioma cases without osseous involvement and direct curative surgery without recurrence. Vice versa, these complex meningioma cases do presumably profit more from PET imaging than “simple, standard” meningioma cases, as remaining or missed meningioma sites, e.g., in the case of an atypical or even anaplastic meningioma, might have a more severe influence on the patient’s outcome. Nonetheless, it must be acknowledged that we did not perform a dedicated volumetric comparison of uptake on [^18^F]SiTATE PET/CT and tumor extension on MRI, as histological workup with an assessment of osseous involvement was not always part of the clinical routine and not the aim of this exploratory study. Nonetheless, further direct comparisons are warranted to further elucidate potential under- or overestimations of tumor boundaries due to the underlying spatial resolution. Within this process of correlating (stereotactic) histopathological specimen with tracer uptake on PET, it would furthermore be interesting to evaluate the contribution of tumor-infiltrating inflammatory cells to the PET signal, especially in the light of new insights of partial macrophage activity/tumor-infiltrating cells in meningiomas [30, 31].

To reach a first preliminary comparison of [^18^F]SiTATE and [^68^Ga]Ga-DOTATOC, we directly compared uptake characteristics in 7 patients with available [^68^Ga]Ga-DOTATOC PET/CT imaging without any specific treatments and without relevant changes on PET imaging with [^18^F]SiTATE. Background activities in the healthy brain, parotid, and pituitary were generally comparable, and partly, slightly higher in [^18^F]SiTATE PET. Meningioma uptake intensity showed a trend towards higher uptake values in [^18^F]SiTATE PET imaging without reaching the level of significance. However, uptake intensities remained within a reasonable range with persistent comparability. Hence, these data from a small subgroup indicate comparable uptake characteristics of [^18^F]SiTATE and [^68^Ga]Ga-DOTATOC. If [^18^F]SiTATE may provide even higher sensitivity for meningioma detection due to the physically better spatial resolution of ^18^F compared to ^68^Ga, a finding that has already been reported for [^18^F]SiTATE PET in patients with neuroendocrine tumors [11–13] needs to be evaluated in direct comparative studies of both tracers.

Given the previously published radiation dose of approximately 0.015 mSv/MBq for [^18^F]SiTATE, this resulted in an approximate overall radiation dose of 2.3 mSv. This represents a slightly lower radiation dose as compared to [^68^Ga]Ga-DOTATOC, [^68^Ga]Ga-DOTATATE, or even ^68^Ga-DOTA-JR11, where former studies reported radiation doses with a range of 0.021–0.026 mSv/MBq [32–34]. Analogously, these findings were also reported for PSMA-targeted PET imaging comparing ^18^F- and ^68^Ga-labeled ligands [35–38]. In line with these data, we also observed a significantly lower radiation exposure in our subgroup analysis directly comparing [^18^F]SiTATE and [^68^Ga]Ga-DOTATOC (2.6 vs. 4.7 mSv).

Besides a lower radiation dose, [^18^F]SiTATE as an ^18^F-labeled SSTR-targeting peptide may provide further advantageous properties. Firstly, a cyclotron-based production with already established radiosynthesis [9, 10] leads to the independence of predominantly costly ^68^Ge/^68^Ga-generators and thus allows for large badge productions of this ^18^F-imaging agent; however, economic aspects of both approaches have to be addressed in dedicated cost-effectiveness analyses and need to take into account individual local factors such as the presence of an on-site cyclotron, availability of personnel for radiolabeling, and a number of patients of interest per day. Depending on the age of the respective generators, only very limited numbers of patients can undergo PET scanning after a batch synthesis on a single day, in most cases, a low one-digit number of patients. Cyclotron-based synthesis allows for significantly higher activities per synthesis and, consequently, a higher number of patients that can be examined per day. Even the transportation of [^18^F]SiTATE to other additional imaging sites is manageable due to the high activity obtained (satellite principle). This logistic advantage is facilitated by ^18^F’s significantly longer half-life compared to ^68^Ga (110 vs. 68 min). Moreover, the significantly lower positron energy of ^18^F compared to ^68^Ga (mean positron energy 0.25 vs. 0.83 MeV) leads to better spatial resolution in PET imaging due to a shorter positron range (mean range 0.6 vs. 3.5 mm), as already demonstrated in patients with neuroendocrine tumors [11–13]. This might be of particular help in meningioma imaging, where delicate structures such as the optic nerve or the skull base might be affected, and the resolution of PET imaging also influences clinical management in terms of resection and radiotherapy planning. However, a general “superiority” of an approach (^68^Ga vs. ^18^F) cannot be stated, as local needs and patient characteristics and patient numbers also play a major role in the management of SSTR-directed imaging, as, of course, the set-up and maintenance of, e.g., an on-site cyclotron also represents a costly facility with the additional need for specialized, well-trained, and experienced personnel.

Besides [^18^F]SiTATE, additional ^18^F-labeled peptides, such as Al^18^F-NOTA-octreotide, demonstrated high clinical feasibility in patients with neuroendocrine tumors [39–45]. However, no data on patients with meningioma are available so far.

Taken together, [^18^F]SiTATE shows excellent contrast for meningioma imaging with comparable and in some cases superior uptake intensities and background activities compared to [^68^Ga]Ga-DOTATOC, but lower radiation exposure and significantly less logistic constraints (longer half-life, large-badge production, established synthesis, etc.) that might foster widespread use of SSTR-targeted PET imaging in neuro-oncology.

Limitations of this study must be addressed; firstly, this is an exploratory analysis evaluating the general clinical feasibility of [^18^F]SiTATE in meningioma patients; hence, no clinical scenario (e.g., sensitivity for tumor tissue at suspected recurrence) is evaluated in detail. Moreover, a histological evaluation of every included case was not available, especially lesions highly suggestive of being benign are scarce. In borderline cases, clinical follow-up was consulted as a clinical surrogate. Here, further studies evaluating specific clinical questions and studies in direct spatial correlation with stereotactic biopsies/multiple biopsies during surgery are currently in preparation to further elucidate the value of this novel ligand in a particular clinical setting to derive specific, quantitative cut-off values to differentiate, e.g., post-operative scar tissues from meningioma recurrence/remnants, as already performed using [^68^Ga]Ga-DOTATATE [46]. Moreover, we conducted a subgroup analysis of seven patients with available [^18^F]SiTATE and [^68^Ga]Ga-DOTATOC PET/CT scans. The aim of this small-size analysis was to get first impressions and quantifications of these two ligands in meningioma patients. However, this does not replace a direct, statistically powered head-to-head comparison of these two tracers, especially regarding certain clinical questions. Also, the impact of the better spatial image resolution of [^18^F]SiTATE on clinical issues in comparison to standard ligands such as [^68^Ga]Ga-DOTATOC needs to be investigated further.

## Conclusion

The novel SSTR-targeting PET ligand [^18^F]SiTATE in meningioma patients demonstrates an excellent contrast to unaffected, healthy structures and non-meningioma lesions and is capable of detecting osseous extension and so far unknown meningioma lesions. Due to the labeling with ^18^F, this new ligand offers substantial advantages over ^68^Ga-standard ligands such as large-badge synthesis accommodating a higher number of patients, logistic advantages (satellite principle), better spatial resolution, and lower radiation exposure. The availability of an ^18^F-labeled SSTR ligand such as [^18^F]SiTATE has strong potential to foster a widespread use of SSTR-targeted PET in neuro-oncology.

## Data Availability

Data may be made available by the corresponding author on reasonable request.
