# Correction to: Next‑generation PET/CT imaging in meningioma—first clinical experiences using the novel SSTR‑targeting peptide [^18^F]SiTATE

**DOI:** 10.1007/s00259-023-06409-8

**Published:** 2023-08-29

**Authors:** Marcus Unterrainer, Sophie C. Kunte, Lena M. Unterrainer, Adrien Holzgreve, Astrid Delker, Simon Lindner, Leonie Beyer, Matthias Brendel, Wolfgang G. Kunz, Michael Winkelmann, Clemens C. Cyran, Jens Ricke, Klaus Jurkschat, Carmen Wängler, Björn Wängler, Ralf Schirrmacher, Claus Belka, Maximilian Niyazi, Joerg‑Christian Tonn, Peter Bartenstein, Nathalie L. Albert

**Affiliations:** 1 Department of Nuclear Medicine, University Hospital, LMU Munich, Munich, Germany; 2 Department of Radiology, University Hospital, LMU Munich, Marchioninistr. 15, 81377 Munich, Germany; 3 German Center for Neurodegenerative Diseases (DZNE), Munich, Germany; 4 Munich Cluster for Systems Neurology (SyNergy), Munich, Germany; 5 Fakultät für Chemie und Chemische Biologie, Technische Universität Dortmund, Dortmund, Germany; 6 Biomedical Chemistry, Department of Clinical Radiology and Nuclear Medicine, Medical Faculty, Mannheim of Heidelberg University, Mannheim, Germany; 7 Molecular Imaging and Radiochemistry, Department of Clinical Radiology and Nuclear Medicine, Medical Faculty, Mannheim of Heidelberg University, Mannheim, Germany; 8 Department of Oncology, Division of Oncological Imaging, University of Alberta, Edmonton, AB Canada; 9 Department of Radiation Oncology, University Hospital, LMU Munich, Munich, Germany; 10 German Cancer Consortium (DKTK), Partner Site Munich, German Cancer Research Center (DKFZ), 69120 Heidelberg, Germany; 11 Bavarian Cancer Research Center (BZKF), Munich, Germany; 12 Department of Neurosurgery, University Hospital, LMU Munich, Munich, Germany

**Correction to: European Journal of Nuclear Medicine and Molecular Imaging**

https://doi.org/10.1007/s00259-023-06315-z

The headings of Figure 4 were corrected after the initial publication.

The original article has been corrected.

